# In Vivo Comparison of the Phenotypic Aspects and Molecular Mechanisms of Two Nephrotoxic Agents, Sodium Fluoride and Uranyl Nitrate

**DOI:** 10.3390/ijerph16071136

**Published:** 2019-03-29

**Authors:** Alice Bontemps, Laurine Conquet, Christelle Elie, Victor Magneron, Céline Gloaguen, Dimitri Kereselidze, Karine Tack, Olivier C. Barbier, Yann Guéguen

**Affiliations:** 1 Institut de Radioprotection et de Sûreté Nucléaire (IRSN), PSE-SANTE/SESANE/LRTOX, 92262 Fontenay-aux-Roses, France ; alice.bontemps@irsn.fr (A.B.); laurine.conquet54@gmail.com (L.C.); christelle.elie@irsn.fr (C.E.); victor.magneron-manpower@irsn.fr (V.M.); celine.gloaguen@irsn.fr (C.G.); dimitri.kereselidze@irsn.fr (D.K.); Karine.TACK@asn.fr (K.T.); 2 Centro de Investigación y de Estudios Avanzados del Instituto Politécnico Nacional, Departamento de Toxicología, Av. IPN No. 2508 Col., San Pedro Zacatenco, México City, CP 07360, Mexico; obarbier@cinvestav.mx; 3 Institut de Radioprotection et de Sûreté Nucléaire (IRSN), PSE-SANTE/SESANE/LRSI, 92262 Fontenay-aux-Roses, France

**Keywords:** uranium, fluoride, kidney, KIM-1, apoptosis, inflammation

## Abstract

Because of their nephrotoxicity and presence in the environment, uranium (U) and fluoride (F) represent risks to the global population. There is a general lack of knowledge regarding the mechanisms of U and F nephrotoxicity and the underlying molecular pathways. The present study aims to compare the threshold of the appearance of renal impairment and to study apoptosis and inflammation as mechanisms of nephrotoxicity. C57BL/6J male mice were intraperitoneally treated with a single dose of U (0, 2, 4 and 5 mg/kg) or F (0, 2, 5, 7.5 and 10 mg/kg) and euthanized 72 h after. Renal phenotypic characteristics and biological mechanisms were evaluated by urine biochemistry, gene/protein expression, enzyme activity, and (immuno)histological analyses. U and F exposures induced nephrotoxicity in a dose-dependent manner, and the highest concentrations induced severe histopathological alterations as well as increased gene expression and urinary excretion of nephrotoxicity biomarkers. KIM-1 gene expression was induced starting at 2 mg/kg U and 7.5 mg/kg F, and this increase in expression was confirmed through in situ detection of this biomarker of nephrotoxicity. Both treatments induced inflammation as evidenced by cell adhesion molecule expression and in situ levels, whereas caspase 3/7-dependent apoptosis was increased only after U treatment. Overall, a single dose of F or U induced histopathologic evidence of nephrotoxicity renal impairment and inflammation in mice with thresholds under 7.5 mg/kg and 4 mg/kg, respectively.

## 1. Introduction

General populations can be exposed to both uranium (U) and fluoride (F) because of their natural and anthropogenic presence in the environment. Occupational and other activities lead to heterogeneous exposures to U from ore mining, milling and processing, whereas F exposure comes mainly from dental hygiene products, pesticides and water sources in some regions. These contaminants can be distributed throughout the body after ingestion, inhalation and transdermal exposure and can alter several organs, including bone, lung, liver, brain, and kidney [1,2,3,4]. Among these organs, the kidney is the most sensitive to U and F [5,6,7].

Both U and F are known as nephrotoxicants at high doses, despite notable chemical differences which makes them interesting to compare. Since U is a radioelement emitting alpha particles and a heavy metal, it can induce radiotoxicity or chemical toxicity, depending on its isotopic composition (enrichment of the most radiotoxic U^235^) [4,6,8]. According to numerous oxidation states, the speciation of U is relatively complex. As a primarily cationic substance, U can form complexes with many anions. However, F only presents chemical toxicity and because of its high electronegativity, its anionic form usually binds with cationic species such as those in the calcium-rich areas of bone [9]. 

Because of its functions in filtration, transport and reabsorption, the kidney is a primary target organ of F and U toxicity; F and U exhibit renal accumulation, specifically in the cortical and juxtaglomerular areas where the proximal tubules are located [10,11,12,13]. Indeed, acute exposure to U induces nephrotoxicity in both humans and animals [6,14] and this impairment is mainly observed in proximal tubule renal epithelial cells [15,16]. After acute exposure of 1 to 5 mg/kg U injected intraperitoneally or intramuscularly, rodents demonstrated tubular necrosis and changes in blood chemistry reflecting severe renal alterations [16,17,18]. Similarly, intravenous acute exposures to F induce kidney damage in rats [19], even at low concentrations [20]. The intravenous administration of F in rats induced renal tissue injury in a dose-dependent manner and the toxic effects of F on the kidney were more pronounced in the proximal tubule than in the glomerular region [12,19]. 

Different mechanisms have been identified in F and U toxicity such as oxidative stress, autophagy, genotoxicity and apoptosis, using in vitro models [21,22,23,24,25]. These mechanisms also seem to be involved in F and U toxicity in vivo in addition to inflammation [26,27,28,29,30,31,32]. Interestingly, the two specific mechanisms of apoptosis and inflammation were implicated in kidney cell toxicity after both U and F exposures in vitro but needed to be demonstrated in vivo. Moreover, it would be interesting to understand whether the inflammatory response could be one of the first signs of U or F nephrotoxicity, subsequently leading to cell death through apoptosis. Therefore, our work aims to compare both U and F in a dose-response study to identify the threshold of the appearance of nephrotoxicity in mice and the underlying mechanisms involved, using functional, structural and mechanistic analysis at the gene, protein and in situ levels.

## 2. Materials and Methods 

### 2.1. Animals 

The study was performed with 8-week-old male C57BL/6J mice provided by Charles River (Saint Germain Nuelles, France), weighing 25 ± 5 g. Animals were housed in groups of six under a 12 h/12 h light-dark cycle (light from 7 a.m. to 7 p.m.) with a constant temperature of 21 ± 1 °C. One week of acclimatization was provided before the beginning of the study. Water and food were supplied ad libitum. Body weight gain was monitored twice per week, before and after a 24-h period in metabolic cages. The study was approved by the IRSN Animal Care Committee #81 and conducted in accordance with French legislation on the protection of animals used for experimental purposes (EC Directive 2010/63/EU and French Decret 2013–118). All experiments were approved by the Ethics Committee #81 and authorized by the French Ministry of Research under the reference APAFIS#9324-2017031613376362 v1 (internal project number P16-05), delivered on 4 July 2017.

### 2.2. Intraperitoneal Injection of Animals with U or F and Urine Collection

U was obtained as uranyl nitrate (UN) (Merck-Prolabo, Fontenay-sous-Bois, France), with a specific activity of 14 × 10^3^ Bq/g and composed of 99.74% ^238^U, 0.26% ^235^U, and 0.001% ^234^U. F was obtained as sodium fluoride (NaF) from Sigma (Saint-Quentin Fallavier, France). The ketamine (Imalgène 1000) and xylazine (5% Rompun) solutions were obtained from Centravet (Dinan, France).

**Figure 1:**
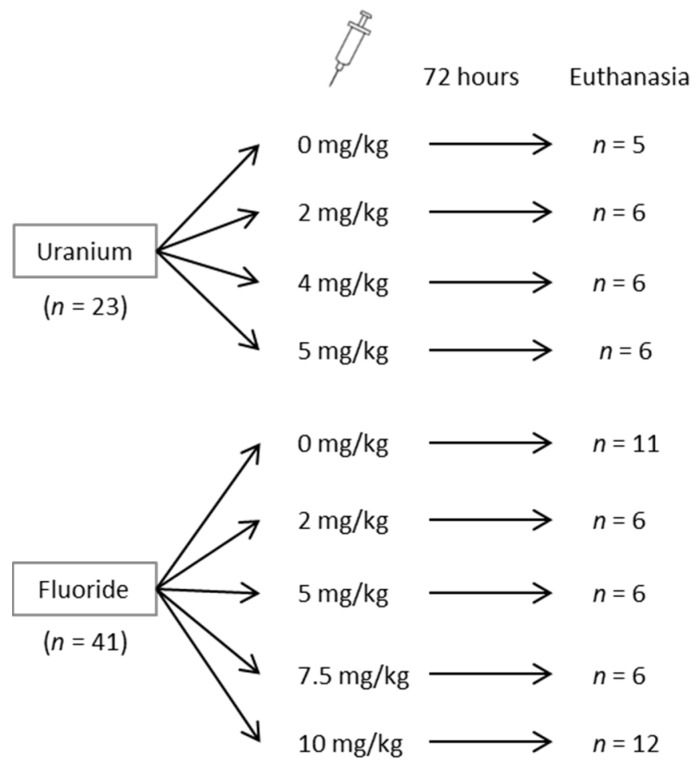
General scheme for the distribution of mice in uranium and fluoride groups and the doses administered.

Mice were divided into two groups as shown in Figure 1: the first group was treated with UN, whereas the second group was treated with NaF. On two occasions, the animals were housed individually in metabolic cages (Tecniplast, Decines Charpieu, France) for a 24-h period to collect urine samples: four days before and immediately after treatment. After collection, urine samples were subsequently centrifuged at 3000 g for 2 min at 4 °C, and the supernatants were isolated and stored at −80 °C. Mice were treated with an intraperitoneal injection with saline solution (NaCl) as a control, or treated with 2, 4 or 5 mg/kg UN (UO_2_(NO_3_)_2_) or 5.5, 11, 16.6 or 22.1 mg/kg NaF (corresponding to 2, 5, 7.5 and 10 mg/kg F). UN and NaF were diluted in a NaCl solution and filtered the day of the injection. Animals were then euthanized 72 h later. The choice of doses and time of exposure was based on literature and previous experiments of the authors showing a peak of renal effects at 72 h post-injection for U [14,16,17,18,19,20]. The experimental design and the animal distribution are presented in Figure 1. 

### 2.3. Euthanasia and Kidney Collection

Seventy-two hours after injection, the animals were anaesthetized by intraperitoneal injection of 100 mg/kg ketamine and 10 mg/kg xylazine and euthanized by exsanguination. Both kidneys were collected, weighed and sagittally cut. Half the right kidney was placed in 4% formaldehyde for histopathological analysis, and the other half was used for U or F quantification. The entire left kidney was designated for biomolecular analysis after being flash-frozen in liquid nitrogen and stored at −80 °C.

### 2.4. U and F Measurements in Urine and Kidney Tissue

U was quantified in urine and kidney by inductively-coupled plasma mass spectrometry (ICP-MS) (ICP-MS, PQ, Excell, Thermo Electron, Villebon-sur-Yvette, France). U was measured in urine after dilution. The half-kidney was digested in nitric acid and hydrogen peroxide, and the samples were then evaporated and dissolved in nitric acid. After the appropriate dilution, the U content was measured using bismuth as an internal control and a U calibration curve. The detection limit of U was determined by ICP-MS: 0.5 ng/L for ^238^U and 0.01 ng/L for ^235^U. F was quantified in urine and kidney by a potentiometric method using an ion selective electrode (Thermo Scientific Orion, Villebon-sur-Yvette, France). The urine samples were diluted before measurements, and the half-kidneys were transferred into platinum crucibles and treated with a 5% magnesium acetate solution. As described by Inkielewicz in 2003, the specimens were pyrolyzed in an oven at 500 °C for 24 h, and the cooled samples were transferred into polyethylene measuring vessels by rinsing the crucibles with perchloric acid solution, a 1 molar solution of citrate and finally total ionic strength adjustment buffer at pH = 5.2 [33].

### 2.5. Urinary Biochemical Measurements

An automated Konelab 20 (Thermo Scientific) was used to assay the levels of creatinine, urea, total proteins, glucose, calcium (Ca), potassium (K), phosphorus (P) and sodium (Na) in urine. All the reagents came from Thermo Electron Corporation (Villebon-sur-Yvette, France).

### 2.6. Histopathology and Immunostaining

After 24 h in 4% formaldehyde, the preserved half-kidney was dehydrated before being embedded in paraffin and cut into 5 µm slices with a microtome. Hematoxylin, eosin, and saffron (HES) staining was conducted, and the slides were sent to an outside expertized laboratory of pathologists to perform the anatomopathological analyses (Biodoxis Laboratories, Romainville, France). Glomerular damage was defined by the presence of glomerulosclerosis and glomerular cystic dilatation, whereas tubular lesions were defined by the following: (1) necrosis; (2) regeneration and dilatation of the tubules; (3) interstitial inflammation; and (4) fibrosis. The different kinds of lesions were scored from 0 to 4 for each animal. The total sum of all lesions corresponded to the global scoring and the percentage of each lesion among the tubulointerstitial lesions was then calculated as previously described [34]. Paraffin-embedded slices were deparaffinized and hydrated in descending gradations of ethanol and in 3% H_2_O_2_ diluted in PBS to block endogenous peroxidase activity. For kidney injury molecule-1 (KIM-1) staining, antigen retrieval was achieved with citrate buffer at pH = 6. Sections were then incubated overnight with anti-KIM-1 (Abcam, Paris, France, ab47635) using a 200-fold dilution at 4 °C in a moist chamber. After washing, the slices were incubated with Alexa Fluor goat anti-rabbit secondary antibody (Abcam, ab150061). Finally, the nuclei were stained with 4′,6-diamidino-2-phenylindole contained in Vectashield antifade mounting medium for fluorescence visualization. Ten photomicrographs per animal were collected with a LEICA DM 4000B fluorescence microscope, and semiquantification of fluorescent labeling was performed with Histolab Software (version 8.1.2, Microvision Instruments, Lisses, France). For intercellular adhesion molecule (ICAM) and vascular cell adhesion molecule (VCAM) staining, antigen retrieval was achieved with Tris/EDTA buffer at pH = 9. Sections were then incubated for 1 h with anti-ICAM-1 (Abcam, ab119871) diluted 250-fold or with anti-VCAM-1 (Abcam, ab134047) diluted 1000-fold at room temperature in a moist chamber. After washing, the slices were incubated with anti-rat and anti-rabbit secondary antibodies to be finally stained using a DAB revelation kit (MMFrance, Brignais, France). A counterstaining with hematoxylin was performed to identify renal structures. Ten photomicrographs per animal were collected with a DM 4000B microscope (LEICA, Paris, France) and semiquantification of stained area was performed using Histolab Software. 

### 2.7. KIM-1 and CLU Detection in Urine

KIM-1 and clusterin (CLU) were measured in urine samples using ELISA kits and according to the manufacturer’s instructions (R&D Systems, Lille, France). To comply with the concentration intervals of the assay, the urine samples were diluted 1:100 for the CLU assay and 1:10 to 1:100 for the KIM-1 assay.

### 2.8. Real-Time RT-PCR

Total RNA was extracted from the renal cortex by following the manufacturer’s instructions (Total RNA isolation kit, Qiagen, Les Ulis, France) and was reverse-transcribed into cDNA with High-capacity cDNA reverse transcription kits (Fisher Scientific, Illkirch, France). Real-time polymerase chain reactions (RT-PCR) was made with a final concentration of 1 ng/μL cDNA for 10 µL per well, containing 2.5% v/v primers (Fisher Scientific, Illkirch, France), 83% v/v SYBR (Fisher Scientific, Illkirch, France), and 14.5% v/v sterile water. Table 1 reports accession number, and primer sequences of each gene used in this study to analyze the mRNA levels of proteins involved in nephrotoxicity and inflammation: KIM-1, CLU, osteopontin (OPN), ICAM-1, and VCAM-1. The AbiPrism 7900 Sequence Detection System (Applied Biosystems, France) was used to detect real-time RT-PCR products. The comparative ΔΔCt method was used to determine relative quantification of each gene expression in comparison with the geometric average of the Ct values of housekeeping genes hypoxanthine-guanine phosphoribosyltransferase (HPRT) and glyceraldehyde-3-phosphate dehydrogenase (GAPDH). Finally, the fold induction for each treated group was calculated relative to the control group (NaCl).

### 2.9. Caspase 3/7 Activity

Pieces of the renal cortex (25 mg) were put into 1.5 mL Eppendorf tubes with 250 µL hypotonic buffer containing 25 mM HEPES, 5 mM MgCl_2_, 1 mM EDTA, 1 mM Pefabloc, 1 µg/mL pepstatin, 1 µg/mL leupeptin, and 1 µg/mL trypsin inhibitor. Then, the pieces were ground with micropestles and centrifuged at 13,000 rpm for 15 min at 4 °C. The supernatants were collected and diluted 50-fold, and the protein concentrations of the supernatants were determined with a Bradford test. Then, sample was adjusted to 1 mg/mL before the determination of caspase activity with a detection kit (Promega, Charbonnières-les-Bains, France, G8091).

### 2.10. Statistical Analyses

Statistical analyses were performed using the SigmaPlot 11.0 software (Systat Software, Inc., Erkrath, Germany), except for the semiquantification of the immunostainings, for which Histolab Software was used as described previously (Microvision Instruments). A one-way analysis of variance (ANOVA) test was used to compare each group, and the Holm-Sidak method was applied for multiple comparisons. For the semiquantification of immunostainings, a general estimating equation (GEE) was performed using the R (version 3.4.4, R Core Team, Boston, MA, USA) and RStudio software packages (version 1.1.423, RStudio Inc., Boston, MA, USA). The level of significance was set at *p* < 0.05.

## 3. Results

### 3.1. Dose-Dependent Increase of F and U in Urine

The F and U contents in mouse urine samples were measured four days before treatment, and immediately after F and U treatments. Table 2 and Table 3 show the F and U levels in urine, and the levels of both increase proportionally to the concentrations injected into animals.

The U content was also quantified in kidney tissue (data not shown) as previously described [11]. In the case of F, the measurements were below the detection limit of the potentiometer. This protocol is adapted from the method Inkielewicz-Stepniak used in rats [34] but the absence of levels above the detection limit is probably due to the small amount of renal tissue available in mice in contrast to rats. However, U showed a dose-dependent increase in the kidneys after treatment; the controls contained approximately 6 ng of U per gram of tissue, whereas the groups treated with 2, 4 or 5 mg/kg UN showed respectively 5, 14 and 30 µg U per gram of tissue (data not shown).

### 3.2. Traditional Nephrotoxicity Biomarkers

**Figure 2:**
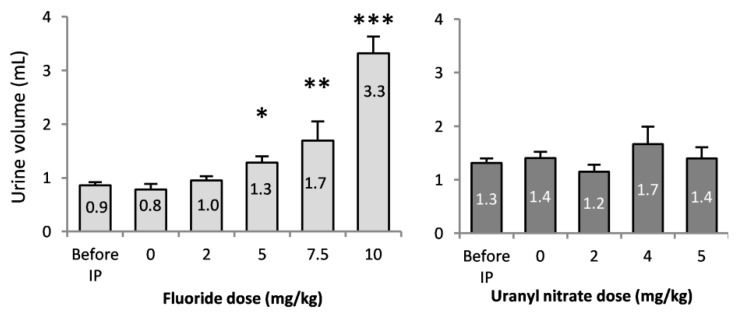
24-h urine volume collected before and immediately after the intraperitoneal injection of 0, 2, 5, 7.5 and 10 mg/kg F or 0, 2, 4 and 5 mg/kg UN. The results are presented as the mean ± standard error of the mean. The asterisk represents a significant difference between the treated (*n* = 6 to 12) and control (*n* = 10) groups (Holm-Sidak test, * *p* < 0.05, ** *p* < 0.005, *** *p* < 0.001).

The diuresis of mice was monitored with metabolic cages before and after treatments (Figure 2). Before treatment, no difference in diuresis among the groups was observed, and animals produced 1.0 ± 0.1 mL of urine in a 24-h period. The injections of 5, 7.5 and 10 mg/kg NaF induced the production of 1.3, 1.7 and 3.3 mL of urine, respectively, in mice, which were significantly different levels of urine production from that of the control. Additionally, the urine levels produced by the groups treated with 7.5 and 10 mg/kg NaF were significantly different (*p* < 0.001), which demonstrates a dose-dependent response to NaF. UN did not show significant differences in urinary flow rate after the injections under our experimental conditions.

Kidney integrity was evaluated by the functional parameters as shown in Table 4 and Table 5. The NaF treatment groups showed an increase in Na and K excretion at 10 mg/kg (*p* < 0.001), suggesting damaged interstitial tissue and renal failure. Glycosuria increased significantly in NaF-injected mice compared to the control mice, which confirms damage to tubular structures. Before treatment, the glucose excreted in urine was approximately 3 µmol/24 h. Mice treated with 10 mg/kg NaF had a 52-fold increase in glycosuria after 24 h. Similarly, UN induced an increase in glycosuria after an injection of 5 mg/kg (*p* < 0.001), but it also induced calciuria starting at 2 mg/kg in the same proportion for all UN treatments (*p* < 0.05).

Taken together, these data show that NaF injections induce diuresis, increased Na and K urinary levels, and glycosuria in a dose-dependent manner. These late nephrotoxicity markers suggest an established nephrotoxicity at 10 mg/kg NaF, whereas UN injections induce the urinary excretion of Ca starting at 2 mg/kg and glycosuria only at 5 mg/kg, without the induction of diuresis. These results do not allow us to conclude the nephrotoxicity of UN under our conditions with the selected biomarkers.

### 3.3. Both U and F Induce Structural Lesions in the Kidney 

**Figure 3:**
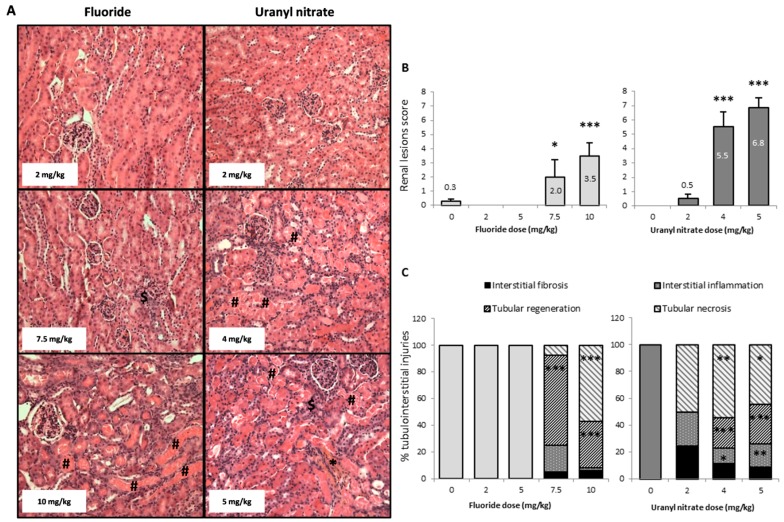
(**A**) Representative photomicrographs (200×) of renal lesions after HES staining in mice 72 h following the intraperitoneal injection of 2, 7.5 and 10 mg/kg F or 2, 4 and 5 mg/kg UN. The sharp symbol (#) indicates mild coagulative necrosis of the tubular epithelium. Affected tubule lumens are filled with hypereosinophilic granular debris. The dollar symbol ($) indicates tubular regeneration with basophilic tubules. The asterisk (*) shows interstitial fibrosis and inflammation, as revealed by saffron coloration. (**B**) The renal lesion scores and (**C**) the percentage of tubular and interstitial injuries (tubular necrosis, basophilic tubules, interstitial inflammation and interstitial fibrosis) in kidneys 72 h after the intraperitoneal injection of 0, 2, 5, 7.5 and 10 mg/kg F or 0, 2, 4 and 5 mg/kg UN. The results are presented as the mean ± standard error of the mean. The asterisk represents a significant difference between the treated (*n* = 6 to 12) and control (*n* = 11) groups (Holm-Sidak test, * *p* < 0.05, ** *p* < 0.005, *** *p* < 0.001).

Figure 3A shows representative photomicrographs of mouse kidneys 72 h after injection. This panel shows groups of mice treated with NaF (2, 7.5, and 10 mg/kg) and UN (2, 4 and 5 mg/kg). A score was established based on the microscopic evaluation of the kidney (Figure 3B) to scale the structural damage from nonexistent to severe levels. In addition, the extent of tubular necrosis, tubular regeneration, interstitial inflammation, and interstitial fibrosis was quantified for each experimental group (Figure 3C).

NaF treatment at 7.5 and 10 mg/kg induced global kidney lesions that were scored as 2 (mild damage) and 3.5 (moderate damage), respectively. However, 4 and 5 mg/kg UN induced scores of 5.5 and 6.8, respectively, corresponding to severe damage (Figure 3B). The NaF injections induced structural damage with tubular necrosis and tissue regeneration at a concentration of 7.5 mg/kg, but no damage was induced at 2 and 5 mg/kg. Moreover, UN induced significant structural damage beginning at 4 mg/kg with tubular necrosis and regeneration in addition to interstitial inflammation.

The histograms shown in Figure 3C represent the extent of the different mechanisms responsible for the tubulo-interstitial injuries in different groups of mice. Thus, 68 and 35% of the tubules presented tubular regeneration 72 h after NaF injections at doses of 7.5 and 10 mg/kg, respectively, and 57% of the tubules showed necrosis after a 10 mg/kg injection. In the UN-treated animals, 2 mg/kg did not induce any apparent tubulo-interstitial injury, whereas 4 and 5 mg/kg induced similar damage to NaF, with approximatively 50% tubular necrosis and 26% tubular regeneration. The UN treatments of 4 and 5 mg/kg also induced significant 12 and 18% increases in inflammation respectively (Figure 3C).

Overall, we can observe distinct histological alterations induced by F and U. On the one hand, an increase in NaF concentrations induces increasing damages in the renal cortex, with the appearance of necrosis at 10 mg/kg, whereas at 7.5 mg/kg we can detect tubular regeneration, a compensation mechanism testifying of an early appearing nephrotoxicity. On the other hand, UN directly induced tubular regeneration and necrosis starting at 4 mg/kg, suggesting that the compensatory mechanism of tubular regeneration may be significantly induced between 2 and 4 mg/kg, before the appearance of irreversible damage.

### 3.4. Biomarkers of Early Kidney Injury Increase after F and U Treatments

**Figure 4:**
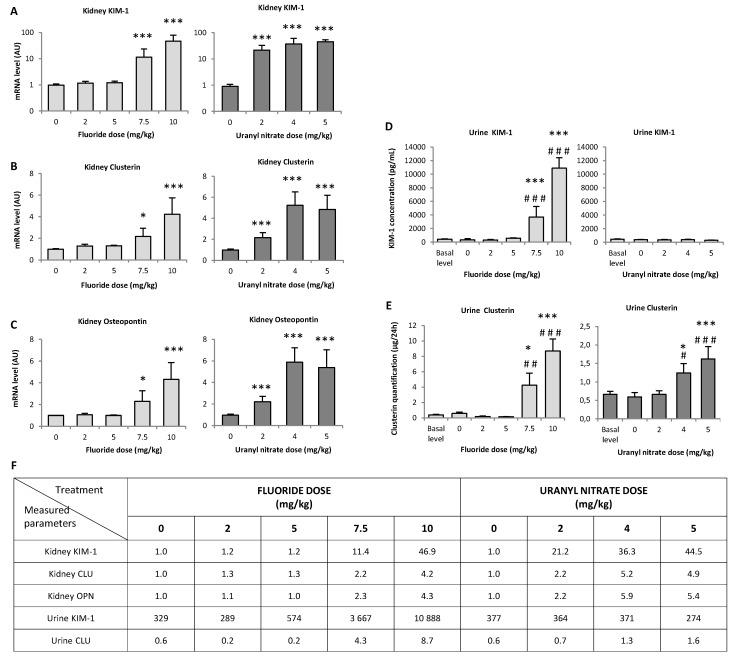
(**A**–**C**) Gene expression of sensitive nephrotoxicity markers in the renal cortex 72 h after the intraperitoneal injection of 0, 2, 5, 7.5 and 10 mg/kg F or 0, 2, 4 and 5 mg/kg UN and (**D**,**E**) urinary protein levels of KIM-1 and clusterin in F- or U-injected mice. (**F**) The measured parameter values are reported in a table with the corresponding units given in each graph. The results of mRNA are expressed as a ratio to the mRNA levels of the housekeeping genes glyceraldehyde 3-phosphate dehydrogenase (GAPDH) and hypoxanthine-guanine phosphoribosyltransferase (HPRT). The results of (A) KIM-1 gene expression are presented on a logarithmic scale. All results are presented as the mean ± standard error of the mean. The asterisk represents a significant difference between the treated (*n* = 6) and control (*n* = 5) groups (Holm-Sidak test, * *p* < 0.05, ** *p* < 0.005, *** *p* < 0.001). The sharp symbol represents a significant difference between the treated (*n* = 6) and preinjected (*n* = 41 to 47) mice (Holm-Sidak test, ^#^ *p* < 0.05, ^##^ *p* < 0.005, ^###^ *p* < 0.001).

KIM-1 is a protein that is overexpressed in the early stages of kidney damage. In the renal tissue of animals treated with 7.5 and 10 mg/kg NaF, KIM-1 gene expression was enhanced by 11- and 47-fold, respectively, compared to the control (*p* < 0.001); however, 2, 4 and 5 mg/kg UN induced 21-, 36- and 44-fold increases in KIM-1 gene expression, respectively (*p* < 0.001) (Figure 4A). As expected, KIM-1 gene expression seems to increase with increasing doses of both treatments: 10 mg/kg NaF induced increased KIM-1 expression compared to 7.5 mg/kg NaF (*p* < 0.05), while UN increased KIM-1 expression at the concentrations between 4 and 5 mg/kg (*p* < 0.05).

The gene overexpression of KIM-1 in tissues was confirmed by the secretion of KIM-1 in urine samples after NaF treatment, whereas KIM-1 secretion was not increased in urine samples after UN treatment (Figure 4D). Specifically, in the case of NaF, the urinary levels of KIM-1 significantly increased in dose-dependent manner: *p* < 0.001 for 7.5 vs. 5 mg/kg and *p* < 0.05 for 7.5 vs. 10 mg/kg. 

**Figure 5:**
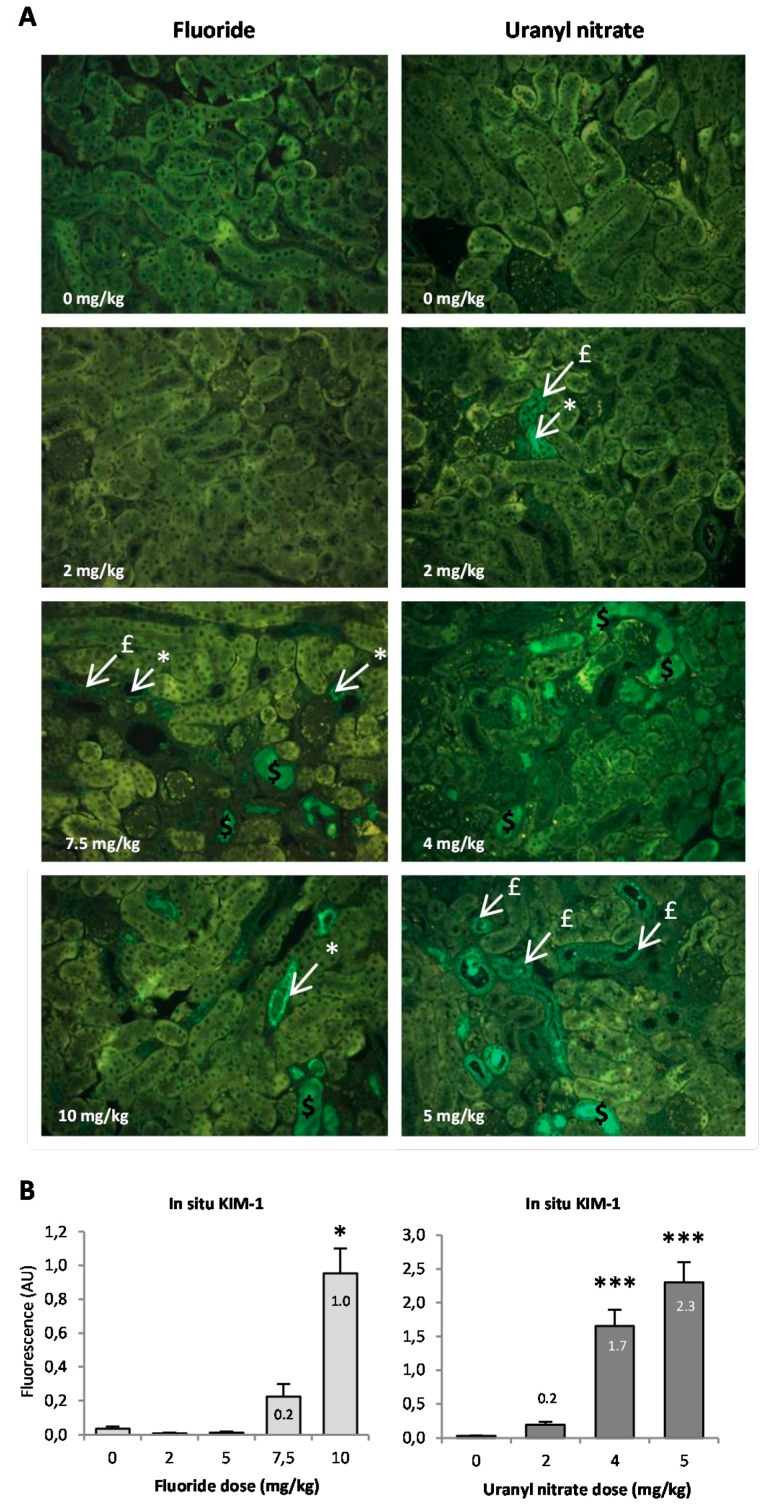
(**A**) Representative photomicrographs (200×) of KIM-1 in situ expression in the kidney of mice injected with F (0, 2, 5, 7.5 and 10 mg/kg) or U (0, 2, 4 and 5 mg/kg). The dollar symbol ($) indicates the expression of KIM-1 in the lumen of proximal tubules. The sharp symbol (#) indicates KIM-1 expression in tubular cells. The asterisk indicates KIM-1 expression in the apical pole of tubular cells. (**B**) The results of the semi-quantification of KIM-1 in situ expression are presented as the mean ± standard error of the mean. The asterisk represents a significant difference between the treated (*n* = 6) and control (*n* = 5) groups (general estimating equation, * *p* < 0.05, ** *p* < 0.005, *** *p* < 0.001).

The in situ detection of KIM-1 in kidney slides is shown in Figure 5A; we can identify KIM-1 expression in the apical pole of tubular cells, distinguish the site of the synthesis of the protein (indicated by white arrows) from its expression in the whole tubular cells (indicated by red arrows) and visualize KIM-1 expression in the lumen after secretion (indicated by orange arrows). Using the photomicrographs, we observed that KIM-1 is absent from the lumen 72 h after a 2 mg/kg UN injection, whereas KIM-1 expression in the lumen is noticeable starting at 7.5 mg/kg NaF and 4 mg/kg UN. KIM-1 in situ expression was quantified (Figure 5B), and under our experimental conditions, the protein appeared to be significantly upregulated starting at 10 mg/kg NaF (*p* < 0.05) and 4 mg/kg UN (*p* < 0.001). The highest concentrations of NaF and UN treatments induced 23.8- and 76.7-fold increases in KIM-1, respectively, compared to their control groups. These results, in addition to the gene expression and urinary secretion of the protein, indicate that KIM-1 increases in proportion to NaF treatment starting at 7.5 mg/kg, whereas UN does not seem to induce KIM-1 secretion in urine but does induce its gene and in situ expression in the kidney.

Similarly, OPN and CLU, proteins considered early biomarkers for a wide variety of acute or chronic renal impairments, were analyzed. In kidney extracts, OPN gene expression was increased by 2.3- and 4.3-fold after the 7.5 (*p* < 0.05) and 10 mg/kg (*p* < 0.001) NaF injections, respectively, whereas OPN was increased by 2.2-fold after a 2 mg/kg UN injection and by 5.5-fold after exposure to 4 or 5 mg/kg UN (*p* < 0.001) (Figure 4C). CLU gene expression increased to a similar extent after NaF and UN injections in mice (Figure 4B), and the protein level was increased by 2.4- and 4.4-fold in urine samples after 7.5 (*p* < 0.05) and 10 mg/kg (*p* < 0.001) NaF injections, respectively. However, 4 and 5 mg/kg UN induced 2.2-fold (*p* < 0.05) and 2.7-fold (*p* < 0.001) increases in the CLU in urines, respectively (Figure 4E). Similar to KIM-1, CLU secretion in urine was lower in UN-treated mice than in NaF-treated mice when comparing the initial gene expression in kidney.

Overall, it seems that the gene expression of the nephrotoxicity biomarkers CLU and OPN and the secretion of CLU in urine samples reached a plateau starting at the 4 mg/kg UN injections, whereas NaF treatments showed an increase between 7.5 and 10 mg/kg for all biomarkers (genes, protein secretion and in situ levels). According to our results, the threshold of appearance of these nephrotoxicity biomarkers must be between 5 and 7.5 mg/kg NaF and between 2 and 4 mg/kg UN as determined by protein levels in urines and in situ.

### 3.5. ICAM and VCAM Increase with F and U Treatments 

**Figure 6:**
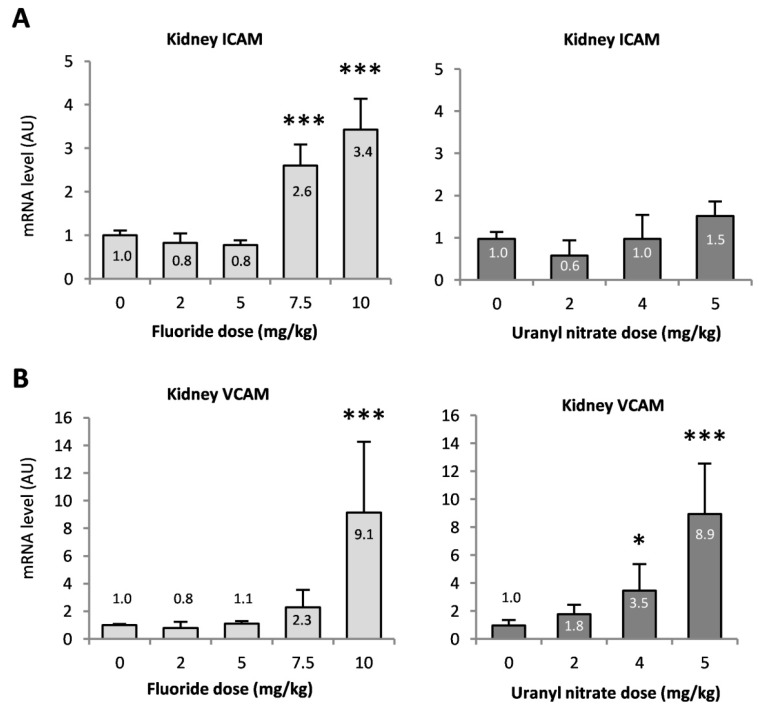
Gene expression of the inflammatory markers (**A**) ICAM and (**B**) VCAM in the renal cortex 72 h after intraperitoneal injection of 0, 2, 5, 7.5 and 10 mg/kg F or 0, 2, 4 and 5 mg/kg UN in mice. The mRNA results are expressed as a ratio to the mRNA levels of the housekeeping genes glyceraldehyde 3-phosphate dehydrogenase (GAPDH) and hypoxanthine-guanine phosphoribosyltransferase (HPRT). The results are presented as the mean ± standard error of the mean. The asterisk represents a significant difference between the treated (*n* = 6) and control (*n* = 5) groups (Holm-Sidak test, * *p* < 0.05, ** *p* < 0.005, *** *p* < 0.001).

ICAM-1 and VCAM-1 are two adhesion molecules involved in the recruitment of inflammatory cells. ICAM gene expression was mostly enhanced after NaF exposure, with 2.6- and 3.4-fold increases after the 7.5 and 10 mg/kg injections, respectively (*p* < 0.001). The expression of the VCAM gene was also increased by 2.3- and 9.1-fold at 7.5 and 10 mg/kg NaF (*p* < 0.001 at 10 mg/kg), respectively, whereas UN treatment increased VCAM gene expression by 3.5- and 9-fold after 4 (*p* < 0.05) and 5 mg/kg (*p* < 0.001) UN injections compared to the control, respectively (Figure 6).

**Figure 7:**
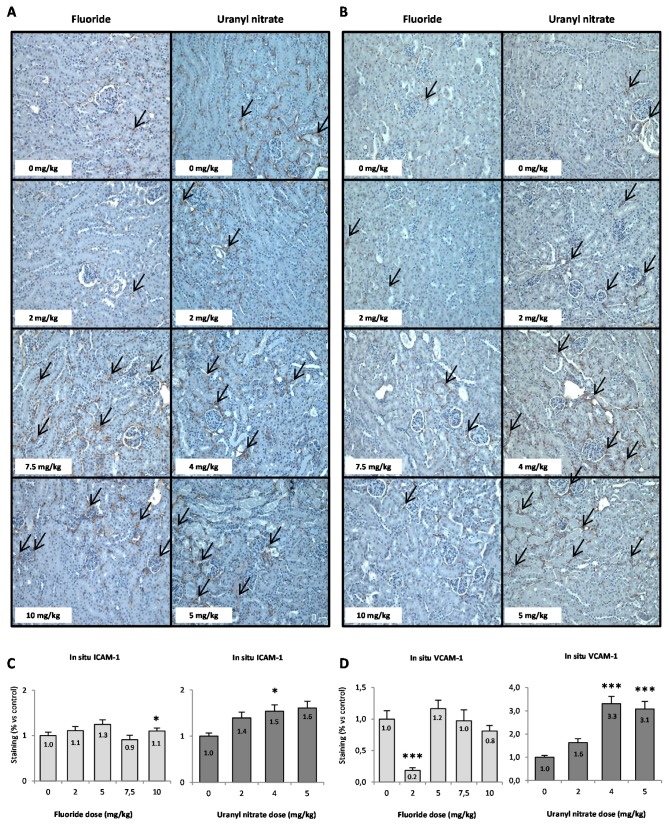
(**A**) Representative photomicrographs (200×) of ICAM (**A**) and VCAM (**B**) in situ expression in the kidneys of mice injected with F (0, 2, 5, 7.5 and 10 mg/kg) or UN (0, 2, 4 and 5 mg/kg). Black arrows indicate the presence of CAM proteins in the renal cortex. The results of the semi-quantification analyses of (**C**) ICAM and (**D**) VCAM in situ expressions are presented as the mean ± standard error of the mean. The asterisk represents a significant difference between the treated (*n* = 6) and control (*n* = 5) groups (general estimating equation, * *p* < 0.05, ** *p* < 0.005, *** *p* < 0.001).

The in situ presence of ICAM and VCAM proteins is shown in Figure 7A,B, and the semi-quantifications of their level in the renal cortex are evaluated in Figure 7C,D. The semi-quantification of VCAM (Figure 7D) shows that exposure to 2 mg/kg NaF induced a downregulation of VCAM, suggesting a possible anti-inflammatory effect at low doses of NaF. There was no significant increase in ICAM and VCAM regulation in situ after acute treatment with NaF, but UN did induce VCAM upregulation starting at 4 mg/kg (*p* < 0.001).

These results show that the in situ levels of ICAM and VCAM confirm the gene expression levels observed after UN treatment, whereas the increase in gene expression after NaF treatment was not observed under our conditions. In the case of UN, the results shown in Figure 6 and Figure 7D suggest the involvement of the molecule VCAM in the recruitment of pro-inflammatory cells and could allow us to identify inflammation as a possible mechanism of U nephrotoxicity.

### 3.6. Caspase 3/7 Activities in the Kidneys of Mice Treated with F or U

**Figure 8:**
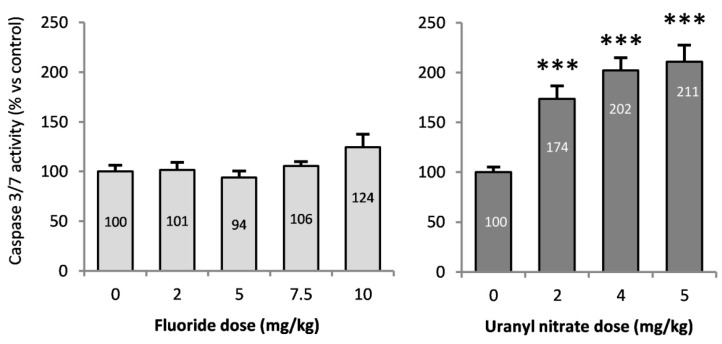
Caspase 3/7 activity in the renal cortex of mice 72 h after intraperitoneal injection of 0, 2, 5, 7.5 and 10 mg/kg F or 0, 2, 4 and 5 mg/kg UN. The results are presented as the mean ± standard error of the mean. The asterisk represents a significant difference between the treated (*n* = 6) and control (*n* = 5) groups (Holm-Sidak test, * *p* < 0.05, ** *p* < 0.005, *** *p* < 0.001).

Caspases 3 and 7 are effector proteins of apoptosis, and an increase in their activities implies the induction of this mechanism of death. Caspase 3/7 activity was evaluated in the renal cortex (Figure 8) and exhibited a significant increase beginning at 2 mg/kg UN (*p* < 0.001), with a 210% increase after a 5 mg/kg injection (compared to control), whereas NaF did not significantly induce apoptosis. Caspase-dependent apoptosis seems to be induced starting at 2 mg/kg UN without any difference between treatment doses.

## 4. Discussion

The kidney is a common target of U and F toxicity, and the comparison of their in vivo biological effects would help to elucidate their nephrotoxic molecular mechanisms. The goal of this study was to determine the threshold of nephrotoxicity using biomarkers sensitive to changes specifically in the kidney and to determine the role of inflammation and apoptosis in the renal effects of F- or U-induced nephrotoxicity in mice. Thus, groups of mice were treated with either NaF or UN at a range of toxic and nontoxic doses derived from the literature. Renal biological effects were analyzed 72 h later, a time previously described as the delay of appearance of the peak of nephrotoxicity [18]. The urinary concentrations of F and U were comparable to the urinary concentration of F after 40 days of exposure to 15–50 mg/L NaF through drinking water in rats [35] or the renal tissue content of U in rats treated with similar levels of U (2–5 mg/kg) [36]. Regardless of the dose used in our study, neither NaF nor UN induced weight loss, but starting at a dose of 5 mg/kg, NaF induced diuresis in a dose-dependent manner (Figure 2), as previously described [20]. NaF and UN also induced significant glycosuria, suggesting damage to tubular integrity. These changes in traditional parameters of nephrotoxicity suggest moderate or severe kidney damage induced by UN or NaF treatment starting at 5 and 10 mg/kg, respectively. The renal histopathology study confirmed that the NaF and UN treatments induce a significant increase in tubular damage and allowed us to determine a threshold of appearance for each kind of renal injury. Interestingly, the highest concentrations of NaF and UN induced approximately the same level of necrosis in the tubulointerstitial area and tubular regeneration (Figure 3). These results are in accordance with previous studies showing the induction of necrosis after acute treatments of F or U in other in vivo models [17,37]. Inflammation, necrosis and regenerative processes were detected and quantified by histopathological scores. These types of lesions were also found in previous studies [18,38,39,40,41] and sometimes with glomerular damage too [17,42]; however, no structural lesions were previously observed in the kidney after acute F treatment [43]. Under our experimental conditions, the tubular regeneration with epithelial proliferation and tubular vacuolization and distortion induced in mice treated with 7.5 mg/kg NaF showed that kidney injuries may be reversible at this level, whereas the necrosis at 10 mg/kg suggests irreversible kidney impairment [44,45]. Indeed, previous studies showed a link between tubular regeneration and kidney recovery after gentamicin treatment [46] or 28 days after an injection of 4 mg/kg UN in mice showing partial recovery [18]. Accordingly, a proteomic analysis showed that tubular regeneration also occurred in chronic fluorosis in rats exposed to 221 mg/L NaF for 8 weeks, with the overexpression of proliferation-related proteins in renal tissue [47].

To more precisely evaluate the kidney injuries induced by the NaF and UN treatments, early biomarkers of kidney disease were measured in situ (Figure 5) and by analyzing gene expression (Figure 4). KIM-1 is a type 1 transmembrane protein whose expression is markedly upregulated in proximal tubule injuries [35,48,49]. In our experimental models, KIM-1 gene expression in the kidney significantly increased beginning at 7.5 mg/kg NaF and 2 mg/kg UN, respectively. For NaF, a similar sensitivity was obtained when the histopathology parameters were evaluated. For UN, the lesion score was not significant at 2 mg/kg; thus, a better threshold of nephrotoxicity was detected using the sensitive biomarker KIM-1 than that obtained using histopathology. The in situ expression level of KIM-1 confirmed a tubular injury starting at 7.5 mg/kg NaF and 2 mg/kg UN and revealed different stages of tubular alteration. Indeed, KIM-1 is a transmembrane protein that is mainly expressed in the apical pole of epithelial cells starting from minimal or mild tubular injury. Its expression throughout tubular epithelial cells and its excretion in the lumen of tubules are correlated with an increase in renal impairment [48,50]. To our knowledge, only two recent papers have studied the induction of KIM-1 in situ after U exposure in male Sprague-Dawley rats, and they showed an increase in KIM-1 in situ levels 48 h after an injection of 4 mg/kg UA [51]. Considering F exposure, if KIM-1 expression has been examined after experimental subchronic exposure to F, it was not previously studied after acute exposure in vivo. In contrast, chronic exposure to UN showed no increase in KIM-1 compared to the control group, even at the concentration of 600 mg/L [11,34] whereas F did after 50 mg/L contamination in drinking water [35].

The overexpression of OPN, a marker of both proximal and distal tubule injury, is also induced after treatments with UN and NaF. However, the extent of the effect and sensitivity of this assay was lower than the KIM-1 analyses. In comparison, subchronic exposure to 50 mg/L F did not induce a significant increase in OPN expression [35], whereas acute exposure to 5 mg/kg UA induced a 2.5-fold increase in its gene expression 72 h after intravenous injection [52]. CLU is a tubular damage marker, and it was overexpressed in the kidney under our experimental conditions. Interestingly, even if CLU gene expression was similarly induced after high treatments of NaF or UN (approximately 4.5-fold increase), the CLU urinary levels were not with the same order of magnitude for NaF (8.7-fold increase) and UN (1.6-fold increase). Similarly, KIM-1 was not induced in the same order of magnitude in the kidney and urine samples after UN treatment, unlike NaF. Yet, previous studies have shown that a good correlation between KIM-1 or CLU mRNA levels in the kidneys and urinary levels existed [34,53,54]. The differences observed in our conditions between the kidney and urinary levels are very likely due to the differences of timing of analysis. Indeed, the urine samples were collected directly after treatment, whereas the kidneys were removed 72 h after treatment. Interestingly, this difference of timing does not seem to have an impact on F nephrotoxicity evaluation, whereas it does impact the U analysis. These results could indicate a difference between the progressions of the U- and F-induced renal injury: U would have a slower mechanism of nephrotoxicity which is not identified on a 24-h period post treatment whereas F would have an immediate acute nephrotoxicity after the exposure. However, our data allow us to conclude that KIM-1 is a more sensitive biomarker than OPN and CLU for F and U nephrotoxicity detection. The histopathological study and the scoring suggested that NaF induced less injury than UN, but the molecular study demonstrated that more specific biomarkers, such as KIM-1, allow us to precisely quantify the renal damage induced by UN or NaF on proximal tubular epithelial cells.

In addition, KIM-1 may play a role in the regulation of the molecular mechanisms of nephrotoxicity. In fact, the involvement of KIM-1 in the phagocytosis of dead cells from the tubular lumen has been identified [55,56]. Participating in the clearance of apoptotic debris of the tubular lumen, KIM-1 confers protection to the kidney after an acute tubular injury. Under our conditions, UN induced apoptosis after a 72-h period but not after 48 h (data not shown); thus we hypothesize that KIM-1 participates in the protection of the kidney during the acute nephrotoxicity induced by UN after a longer recovery period. F-induced apoptosis was observed after acute exposure in different models, but not in the kidney [29,30,31]. In vitro, NaF induces the downregulation of Bcl-2 in human fibroblasts, stimulating the mitochondrial cell death pathway [25]. After acute exposure to NaF in vivo, we did not observe a change in Bcl-2 gene expression (data not shown) or effector proteins of apoptosis caspases 3/7 in the kidney, whereas UN did induce these responses (Figure 8). Previous studies showed that U induces caspase-dependent apoptosis in normal rat kidney proximal cells [22] and that U induces the modulation of apoptosis-related genes in C57BL/6 mouse kidneys after a single dose of 5 mg/kg [57], which is relevant to our results. In contrast, we showed that the main death mechanism induced in acute NaF nephrotoxicity is necrosis, as revealed by histopathology scoring.

In addition to being implied in the clearance of apoptotic debris from the tubular lumen and conferring protection after an acute tubular injury, KIM-1 has also been identified as an inhibitor of innate immunity, inducing a decrease in pro-inflammatory cytokines and an increase in anti-inflammatory growth factor secretion [58]. The renal histopathology highlights the induction of interstitial inflammation after UN treatment at 5 mg/kg, but no inflammation was reported after NaF treatment. Some studies have shown pro-inflammatory gene induction in the kidney and an increase in inflammatory cytokine expression and production in the lungs after U exposures [57,59]. Similarly, F has been linked with inflammation in rat lung tissues and with endothelial dysfunction but these effects have not yet been shown in the kidney [60,61,62]. Our study also focused on the expression of the inflammatory markers ICAM and VCAM after acute in vivo exposure to NaF and UN in the kidney. The CAMs are cell adhesion proteins involved in immune cell recruitment during inflammation [63]. It is known that their expression is elevated in damaged kidneys [46,64,65]. ICAM and VCAM mRNA expression were significantly increased after NaF acute treatment, and ICAM in situ expression in the kidney was slightly increased after 10 mg/kg. Moreover, UN induced VCAM mRNA level in the renal tissue starting at 4 mg/kg UN. The in situ protein level of VCAM was also increased under our experimental conditions. Therefore, NaF and UN seem to induce inflammation as demonstrated by the overexpression of ICAM and VCAM in the kidney.

## 5. Conclusions

Overall, NaF and UN induced nephrotoxicity in mice with thresholds below 7.5 mg/kg NaF and between 2 and 4 mg/kg UN. Indeed, even if a dose of 2 mg/kg UN induces gene expression of nephrotoxicity biomarkers and apoptosis in the kidney, most of our results showed signs of nephrotoxicity at 4 mg/kg UN (HES staining, urinary biomarkers, KIM-1 in situ and inflammation). Moreover, KIM-1 quantification in the kidney seemed to be the most sensitive method to identify the threshold of nephrotoxicity for U and F. As shown by ICAM overexpression and overregulation after NaF treatment, inflammation was identified as a possible mechanism of F nephrotoxicity. Similarly, adhesion molecules were induced in the kidney by UN, but these data could be supplemented by other analyses on inflammatory cells. UN also induced caspase-dependent apoptosis 72 h after treatment, involving another mechanism of nephrotoxicity. The identification of the threshold of acute nephrotoxicity and of its mechanisms may be helpful to have a better understanding of U- and F-induced nephrotoxicity, but our data could be supplemented by other analyses confirming our results or identifying new mechanisms of toxicity.

## Figures and Tables

**Table 1:** Primer sequences of the genes analyzed with real-time RT-PCR.

Gene	Accession Number	Forward	Reverse
CLU	AF182509	TCGGGCATCTGGCATCA	AAGCTCACGGGCGAAGAAC
GAPDH	GU214026	TCCACTCACGGCAAATTCAACG	TAGACTCCACGACATACTCAGC
HPRT	NM_013556	TGCTGCGTCCCCAGACTTTTG	AGATAAGCGACAATCTACCAGAGG
ICAM-1	NM_010493	CACCCCAAGGACCCCAAGGAGAT	CGACGCCGCTCAGAAGAACCAC
KIM-1	BC053400	TTTCAGGCCTCATACTGCTTCTC	TGACCCACCACCCCCTTT
OPN	AF515708	CCCTCGATGTCATCCCTGTT	TTCCGTTGTTGTCCTGATCAGA
VCAM-1	BC029823	TCGCGGTCTTGGGAGCCTCA	TGACCGTGACCGGCTTCCCA

**Table 2:** Fluoride content in urine samples before and directly after intraperitoneal injection.

Fluoride		Mean ± SEM
		µg/24 h
Before injection	Control	2.8 ± 0.4
	2 mg/kg	2.8 ± 0.4
	5 mg/kg	2.5 ± 0.3
	7.5 mg/kg	2.7 ± 0.3
	10 mg/kg	2.4 ± 0.2
After injection	Control	3.0 ± 0.5
	2 mg/kg	15.1 ± 1.2 *
	5 mg/kg	35.5 ± 3.2 ***
	7.5 mg/kg	43.0 ± 5.5 ***
	10 mg/kg	60.7 ± 5.4 ***

* *p* < 0.05, ** *p* < 0.005, *** *p* < 0.001, Holm-Sidak test between the treated and control groups. SEM: standard error of the mean.

**Table 3:** Uranium content in urine samples before and directly after intraperitoneal injection.

Uranyl Nitrate		Mean ± SEM
		ng/24 h
Before injection	Control	17 ± 7
	2 mg/kg	32 ± 8
	4 mg/kg	49 ± 21
	5 mg/kg	40 ± 14
After injection	Control	22 ± 9
	2 mg/kg	3 870 ± 497 *
	4 mg/kg	8 323 ± 2 009 ***
	5 mg/kg	8 599 ± 2 442 ***

* *p* < 0.05, ** *p* < 0.005, *** *p* < 0.001, Holm-Sidak test between the treated and control groups.

**Table 4:** Urinary biochemical parameters of mice injected with fluoride.

Fluoride		Creatinine	Glucose	Potassium	Sodium	Phosphorus	Total Protein	Urea	Calcium
		µmol/24 h	µmol/24 h	mmol/24 h	mmol/24 h	mmol/24 h	mg/24 h	mmol/24 h	µmol/24 h
Before injection	Control	4.07 ± 0.60	2.94 ± 0.45	0.14 ± 0.01	0.13 ± 0.02	0.14 ± 0.03	9.03 ± 1.86	1.67 ± 0.23	4.02 ± 0.66
	2 mg/kg	4.31 ± 0.28	4.98 ± 0.30	0.15 ± 0.01	0.13 ± 0.02	0.11 ± 0.03	8.15 ± 4.03	2.26 ± 0.32	4.74 ± 0.89
	5 mg/kg	4.12 ± 0.27	4.32 ± 0.35	0.14 ± 0.01	0.12 ± 0.01	0.12 ± 0.01	14.73 ± 3.47	2.20 ± 0.19	3.22 ± 0.28
	7.5 mg/kg	3.82 ± 0.44	3.03 ± 0.05	0.15 ± 0.05	0.16 ± 0.01	0.15 ± 0.00	7.80 ± 0.28	1.39 ± 0.27	4.88 ± 0.56
	10 mg/kg	3.53 ± 0.52	3.29 ± 0.56	0.13 ± 0.01	0.10 ± 0.01	0.14 ± 0.02	8.50 ± 1.83	1.61 ± 0.26	5.04 ± 1.32
After injection	Control	3.61 ± 0.51	3.41 ± 0.58	0.13 ± 0.01	0.12 ± 0.01	0.13 ± 0.01	9.15 ± 2.47	1.93 ± 0.39	2.58 ± 0.41
	2 mg/kg	4.19 ± 0.38	4.32 ± 0.86	0.11 ± 0.01	0.11 ± 0.01	0.11 ± 0.01	10.66 ± 3.54	2.06 ± 0.39	3.57 ± 0.84
	5 mg/kg	4.56 ± 0.25	4.43 ± 0.43	0.15 ± 0.01	0.15 ± 0.02	0.13 ± 0.01	17.89 ± 2.30	2.55 ± 0.23	3.16 ± 0.35
	7.5 mg/kg	2.11 ± 0.49	17.52 ± 10.50	0.09 ± 0.02	0.13 ± 0.03	0.11 ± 0.02	3.82 ± 0.71	0.91 ± 0.21	2.79 ± 1.25
	10 mg/kg	4.72 ± 0.59	157.4 ± 46.08 ***	0.23 ± 0.03 ***	0.47 ± 0.09 ***	0.15 ± 0.02	16.88 ± 3.76	1.24 ± 0.17	3.89 ± 0.69

* *p* < 0.05, ** *p* < 0.005, *** *p* < 0.001, Holm-Sidak test between the treated and control groups.

**Table 5:** Urinary biochemical parameters of mice injected with uranium.

Uranyl Nitrate		Creatinine	Glucose	Potassium	Sodium	Phosphorus	Total Protein	Urea	Calcium
		µmol/24 h	µmol/24 h	mmol/24 h	mmol/24 h	mmol/24 h	mg/24 h	mmol/24 h	µmol/24 h
Before injection	Control	2.88 ± 0.52	4.31 ± 0.37	0.14 ± 0.01	0.21 ± 0.05	0.20 ± 0.02	10.05 ± 2.75	1.76 ± 0.27	4.03 ± 0.63
	2 mg/kg	3.11 ± 0.52	4.59 ± 0.39	0.14 ± 0.01	0.20 ± 0.02	0.16 ± 0.01	15.90 ± 3.36	1.48 ± 0.22	3.78 ± 0.25
	4 mg/kg	2.66 ± 0.50	3.88 ± 0.44	0.14 ± 0.01	0.23 ± 0.02	0.18 ± 0.02	10.67 ± 2.11	1.45 ± 0.24	4.13 ± 0.63
	5 mg/kg	3.35 ± 0.43	3.51 ± 0.59	0.15 ± 0.01	0.24 ± 0.03	0.18 ± 0.02	16.65 ± 2.73	1.80 ± 0.28	4.16 ± 0.47
After injection	Control	3.09 ± 0.55	4.50 ± 0.34	0.15 ± 0.01	0.21 ± 0.03	0.22 ± 0.01	16.33 ± 3.84	1.70 ± 0.29	3.71 ± 0.42
	2 mg/kg	2.99 ± 0.38	3.22 ± 0.19	0.13 ± 0.01	0.23 ± 0.02	0.17 ± 0.01	12.20 ± 2.00	1.37 ± 0.22	5.70 ± 0.65 *
	4 mg/kg	3.44 ± 0.35	7.06 ± 2.72	0.14 ± 0.02	0.29 ± 0.07	0.16 ± 0.02	11.31 ± 2.36	1.27 ± 0.29	6.82 ± 1.31 *
	5 mg/kg	3.61 ± 0.25	15.76 ± 3.71 ***	0.14 ± 0.01	0.28 ± 0.08	0.18 ± 0.01	19.36 ± 2.90	1.52 ± 0.21	6.23 ± 0.74 *

* *p* < 0.05, ** *p* < 0.005, *** *p* < 0.001, Holm-Sidak test between the treated and control groups.
